# Development of a Small D-Enantiomeric Alzheimer’s Amyloid-β Binding Peptide Ligand for Future In Vivo Imaging Applications

**DOI:** 10.1371/journal.pone.0041457

**Published:** 2012-07-24

**Authors:** Susanne Aileen Funke, Dirk Bartnik, Julian Marius Glück, Kasia Piorkowska, Katja Wiesehan, Urs Weber, Balazs Gulyas, Christer Halldin, Andrea Pfeifer, Christian Spenger, Andreas Muhs, Dieter Willbold

**Affiliations:** 1 Forschungszentrum Jülich, ICS-6, Jülich, Germany; 2 AC Immune, PSE Building B, EPFL, Lausanne, Switzerland; 3 Psychiatry Section, Department of Clinical Neuroscience, Karolinska Institutet, Stockholm, Sweden; 4 Department of Clinical Science, Intervention and Technology, Karolinska Institutet, Stockholm, Sweden; 5 Prodema Management AG, Bronschhofen, Switzerland; 6 Institut für Physikalische Biologie, Heinrich-Heine-Universität Düsseldorf, Düsseldorf, Germany; Cedars-Sinai Medical Center, Maxine-Dunitz Neurosurgical Institute, United States of America

## Abstract

Alzheimer’s disease (AD) is a devastating disease affecting predominantly the aging population. One of the characteristic pathological hallmarks of AD are neuritic plaques, consisting of amyloid-β peptide (Aβ). While there has been some advancement in diagnostic classification of AD patients according to their clinical severity, no fully reliable method for pre-symptomatic diagnosis of AD is available. To enable such early diagnosis, which will allow the initiation of treatments early in the disease progress, neuroimaging tools are under development, making use of Aβ-binding ligands that can visualize amyloid plaques in the living brain. Here we investigate the properties of a newly designed series of D-enantiomeric peptides which are derivatives of ACI-80, formerly called D1, which was developed to specifically bind aggregated Aβ1–42. We describe ACI-80 derivatives with increased stability and Aβ binding properties, which were characterized using surface plasmon resonance and enzyme-linked immunosorbent assays. The specific interactions of the lead compounds with amyloid plaques were validated by *ex vivo* immunochemistry in transgenic mouse models of AD. The novel compounds showed increased binding affinity and are promising candidates for further development into *in vivo* imaging compounds.

## Introduction

Alzheimer’s disease (AD) is a devastating neurodegenerative disorder and the most common cause of dementia. AD affects 27 million people world-wide with steadily increasing numbers, thereby raising significant economic problems and tremendous personal suffer [1]. The two pathological hallmarks that characterize AD are the presence of intracellular neurofibrillary tangles (NFTs) and extracellular neuritic plaques that can be found *post mortem* in the brains of patients [2], [3], [4]. Neurofibrillary tangles consist of twisted filaments of hyperphosphorylated tau protein [5], whereas plaques are primarily composed of amyloid-β (Aβ) [4], [6], a 39–43 amino acid (aa) peptide derived from the amyloid precursor protein (APP) by proteolytic processing [4], [7]. According to the amyloid cascade hypothesis (16), Aβ peptides and, more specifically, their aggregated forms initiate cellular events leading to the pathologic effects of AD [8], [9].

*Pre mortem,* AD is usually diagnosed after the appearance of symptoms by application of tests for cognitive impairment like the mini-mental status examination (MMSE) or the Alzheimer’s disease assessment scale (ADAS) [10], [11]. However, it is a great challenge to correctly diagnose AD at early presymptomatic stages [12], [13], [14]. Several publications support the finding that plaques start to accumulate 10 to 20 years before clinical symptoms appear, leading to substantial and progressive neuronal loss [3], [15], [16]. Therefore, detection and quantitation of amyloid species in the brains of patients during the course of the disease for early diagnosis of AD and for monitoring AD-treatments is a promising and emerging field in AD research. An efficient tool for presymptomatic characterization of the brain may be imaging approaches making use of amyloid specific ligands and positron emission tomography (PET) [17] or single photon emission computed tomography (SPECT). Currently, only a few amyloid PET ligands have been applied in clinical studies (for review, see ref. [18], [19]). Numerous efforts are devoted to develop new, target-specific imaging agents for the detection of amyloid plaques *in vivo*. To be suitable, they should provide highly specific binding to Aβ aggregates, very selective labeling and efficient brain penetration. Moreover, imaging probes are desired with specificity for Aβ1–42 over other Aβ isoforms.

The present study used a small, specific Aβ1–42 binding peptide comprising solely of D-enantiomeric amino acids, termed “D1” [20], [21], [22], or alternatively, ACI-80 [23], [24]. ACI-80 was identified employing a mirror image phage display selection using aggregated Aβ1–42 as a target. *In vitro*, ACI-80 binds preferentially to aggregated Aβ1–42 with a K_D_ in the submicromolar range, whereas monomers are bound to a much less extent. In brain tissue sections derived from patients that suffered from AD, amyloid plaques and leptomeningeal vessels containing Aβ aggregates were stained specifically with a fluorescence-labeled derivative of ACI-80. Fibrillar deposits derived from other amyloidosis were not labeled by ACI-80 [21], [22], [25]. We also demonstrated *in vivo* and *in vitro* that ACI-80 binds specifically to aggregated Aβ1–42 in the brains of APP/PS1 transgenic mice, where diffuse amyloid-β deposits, which do not contain Aβ1–42, were not stained [20].

**Table 1:** Pyroglutamate content of ACI-80, ACI-80-Kϕ and [^127^I]-ACI-80.

	ACI-80_solid_	ACI-80-Kϕ_solid_	[^127^I]-ACI-80_aqueous solution_
Identified amino acid at the N-terminal position			
glutamine	>91.2%	>94.4%	62.6%
pyroglutamate	<8.8%	<5.6%	37.4%
Molecular weight	1421 Da	1907 Da	1548 Da

Kϕ presents a lysine (K) linked to a fluorescein isothiocyanate (ϕ).

Here, we investigate the properties of several derivatives of ACI-80. The novel compounds showed increased binding affinity and are promising candidates for further development into *in vivo* imaging compounds.

## Materials and Methods

### Peptides

For the list of all investigated D-enantiomeric compounds see Table 1. Aβ1–42 peptide was purchased as reversed phase high performance liquid chromatography purified product (JPT Biotech, Berlin, Germany; or Bachem AG, Bubendorf, Switzerland). Identity was confirmed by matrix assisted laser desorption ionization time of flight mass spectrometry (MALDI-TOF-MS).

### General Method for Synthesis of D-peptide Compounds

The non-fluorinated peptides shown in Table 1 were synthesized by JPT Peptide Technologies GmbH, Berlin, Germany.

**Table 2:** List of investigated D-enantiomeric peptide compounds.

Name of compound	Amino acid sequence	Modification
ACI-80	QSHYRHISPAQV	D1
ACI-80-Kϕ	QSHYRHISPAQVKϕ	D1-Kϕ
ACI-87-Kϕ	QSHYRHISPAQ**K**Kϕ	D1-V12K-Kϕ
[^19^F]-ACI-87-Kϕ	QSHYRHISPAQ**K**K[^19^F]ϕ	D1-V12K-K[^19^F]ϕ
ACI-83- Kϕ	**P**SHYRHISPAQVKϕ	D1-Q1P-Kϕ
ACI-89-Kϕ	**P**SHYRHISPAQ**K-**Kϕ	D1-Q1P-V12K-Kϕ
[^19^F]-ACI-89-Kϕ	**P**SHYRHISPAQ**K**K[^19^F]ϕ	D1-Q1P-V12K-K[^19^F]ϕ
ACI-86- Kϕ	**P**S**F**YRHISPAQVKϕ	D1-Q1P-H3F- Kϕ
ACI-82- Kϕ	SHYRHISPAQVKϕ	D1-Q1X- Kϕ
ACI-88-Kϕ	SHYRHISPAQ**K**Kϕ	D1-Q1X-V12K-Kϕ
[^19^F]-ACI-88-Kϕ	SHYRHISPAQ**K**K[^19^F]ϕ	D1-Q1X-V12K-K[^19^F]ϕ
ACI-85- Kϕ	S**F**YRHISPAQVKϕ	D1-Q1X-H3F-Kϕ
ACI-81	**Z**SHYRHISPAQV	D1-Q1Z
ACI-81- Kϕ	**Z**SHYRHISPAQVKϕ	D1-Q1Z-Kϕ
ACI-84- Kϕ	**Z**S**F**YRHISPAQVKϕ	D1-Q1Z-H3F-Kϕ

Modifications in the original amino acid sequence of ACI-80 are printed in bold. Amino acid residues are given in the one-letter-code. All amino-acids are D-enantiomers. Kϕ presents a lysine (K) linked to a fluorescein isothiocyanate (ϕ).

### Synthesis of ^19^F-D-peptide Compounds

[^19^F]-D-peptide synthesis was performed as described earlier [23]. Briefly, to an aqueous solution of peptide, borate buffer (0.5 M, pH 8.61) was added and the color of the solution changed from yellow to dark orange. Slightly excess amount of [^19^F]-*N*-succinimidyl−4-fluorobenzoate (SFB) in acetonitrile was added into this above solution and the reaction mixture was kept at RT for 10 min. The reaction was monitored by HPLC. The crude product was purified by an analytical HPLC column (3×300 mm, 10 µm, waters) using water with 0.1% (v/v) trifluoroacetic acid (TFA) and an acetonitrile (MeCN) gradient (20% to 50%, v/v) as eluent with a flow rate of 2 ml/min. Retention time of the three reference peptides were from 9 to 12 min at a wavelength at 234 nm. Then the product fraction was collected into a pre-filled slightly basic aqueous solution (40 ml, pH was adjusted by NaOH). This diluted fraction was passed through a C18 Sep-Pak plus cartridge (preconditioned with 10 ml ethanol +10 ml water) and the desired product was eluted with 1 ml of ethanol. The reference compounds were confirmed by LC-MS/MS. The purity and the stability of the products were checked by HPLC.

**Table 3:** Results of the binding assays for ACI-80-Kϕ derivatives to Aβ1–42 fibrils using surface plasmon resonance.

Derivative/Modification	Name	Interaction [%]	Dissociation [%]
D1-Kϕ	ACI-80-Kϕ	used as standard and set to 100%	
D1-V12K-Kϕ	ACI-87-Kϕ	324	443
D1-V12K-K[^19^F]ϕ	[^19^F]-ACI-87-Kϕ	153	67
**D1-Q1P**	**ACI-83**		
D1-Q1P-Kϕ	ACI-83-Kϕ	600	200
D1-Q1P-V12K-Kϕ	ACI-89-Kϕ	518	533
D1-Q1P-V12K-K[^19^F]ϕ	[^19^F]-ACI-89-Kϕ	476	0
D1-Q1P-H3F-Kϕ	ACI-86-Kϕ	21	40
**D1-Q1X**	**ACI-82**		
D1-Q1X-Kϕ	ACI-82-Kϕ	393	300
D1-Q1X-V12K-Kϕ	ACI-88-Kϕ	365	667
D1-Q1X-V12K-K[^19^F]ϕ	[^19^F]-ACI-88-Kϕ	294	1233
D1-Q1X-H3F-Kϕ	ACI-85-Kϕ	229	40
**D1-Q1Z**	**ACI-81**		
D1-Q1Z-Kϕ	ACI-81-Kϕ	129	40
D1-Q1Z-H3F-Kϕ	ACI-84-Kϕ	93	40

Interaction and dissociation was measured with respect to the maximal interaction signal during injection and the response 60 s after the end of injection, respectively. ACI-80-Kϕ binding was defined as 100%. Kϕ presents a lysine (K) linked to a fluorescein isothiocyanate (ϕ).

**Figure 1:**
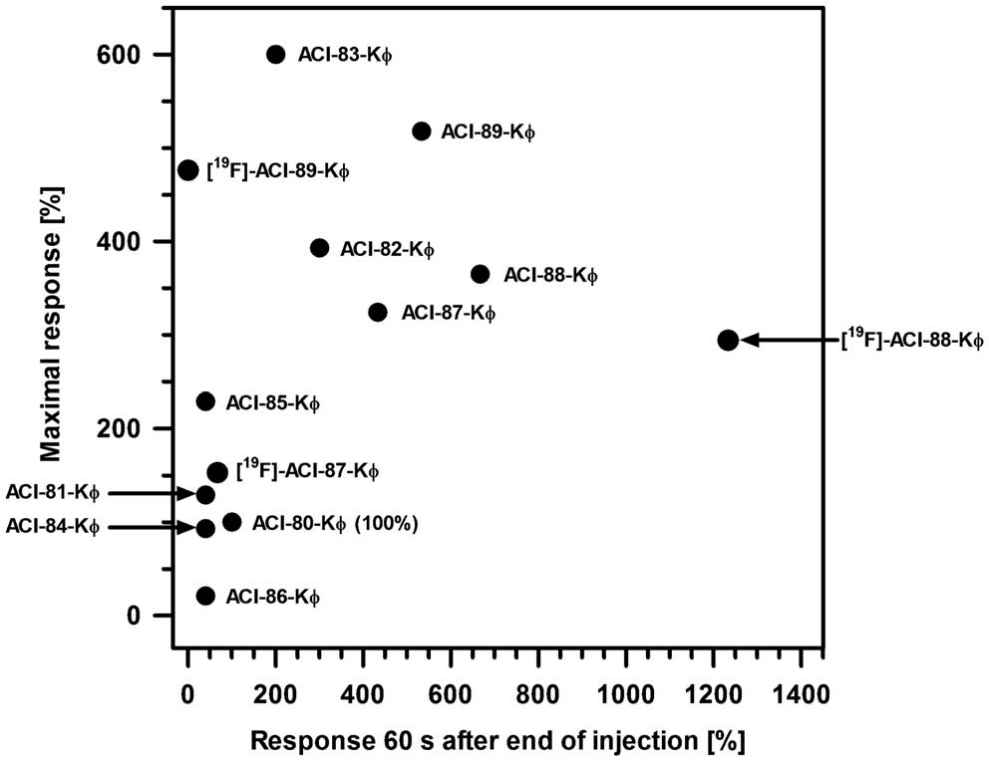
Surface plasmon resonance analysis of the interaction between immobilized Aβ1–42 fibrils and ACI-80-Kϕ and various derivatives (Kϕ presents a lysine (K) covalently linked to a fluorescein isothiocyanate (ϕ). ACI-80 derivatives were solved in running buffer (PBS, pH 7.4). The injected volume of ACI-80 derivatives was 10 µl of a 50 µg/ml concentration using a flow rate of 5 µl/min. The response of ACI-80-Kϕ in resonance units [RU] was defined as 100%. Values >100% denote increased Aβ interaction of the ACI-80 derivative in comparison to ACI-80-Kϕ. All derivatives were ϕ-labeled. Only the variations in comparison to ACI-80-Kϕ are indicated in the figure.

### Analysis of ACI-80 Stability

In solution, N-terminal glutamine peptides such as D1 are prone to convert into N-terminal pyroglutamate species: *Gln-SHYRHISPAQV → Pyr-SHYRHISPAQV*. Therefore, the stability of solid ACI-80, solid ACI-80-Kϕ (ϕ: fluorescein isothiocyanate, covalently linked to the peptide via a lysine (K)) and [^127^I]-ACI-80 in solution was investigated by JPT Peptide Technologies GmbH, Berlin, Germany using HPLC/ESI-MS whereby the relative content of N-terminal Gln peptide and N-terminal Pyr peptide was assessed.

### Surface Plasmon Resonance (SPR)

Aβ1–42 was dissolved in hexafluoroisopropanol (HFIP). After overnight incubation, HFIP was removed by evaporation. The Aβ1–42 film was dissolved in PBS buffer pH 7.4 to a concentration of 1 mg/ml and incubated for 7 days at 37°C.

For the measurements, a Biacore 1000 (GE Healthcare) instrument was used. Aβ1–42 fibrils (6800 RU) were immobilized on a CM5 sensorchip (GE Healthcare) via amine coupling. The running and sample buffer was PBS, pH 7.4. To allow comparison between the ACI-80 compounds, but to avoid potential over-interpretation of the data by fitting multiple *k*_on_ and *k*_off_ values for a yet undefined number of different binding sites, only two values have been taken for further evaluation. Response units achieved under identical injection conditions allow comparison of binding strengths of the compounds. Dissociation rates among the compounds have been compared by measuring the remaining response units 60 s after end of injection as a measure for dissociation (*k*_off_). All measurements have been carried out using the same flow cells with identical concentrations and injection conditions. ACI-80-Kϕ derivatives were injected as analytes in a concentration of 50 µg/ml at a flow rate of 5 µl/min for 2 minutes at ambient temperature. The data were evaluated using BiaEvaluation 4.1. The interactions between Aβ1–42 and ACI-80-Kϕ derivatives are given in resonance units (RU) and in % of ACI-80-Kϕ response units.

**Table 4:** ELISA: Mean binding values for compounds with concentration of 10 µg/ml.

Modification	Nomenclature	Binding values and % binding relative to ACI-80-Kϕ for 10 ug/mL compoundconcentration			
		binding Aβ1–42 monomers		binding Aβ1–42 fibrils	
			%		%
D1-Kϕ	ACI-80-Kϕ	0.49	100	0.90	100
D1-V12K-Kϕ	ACI-87-Kϕ	*0.79	122	*0.93	103
D1-V12K-K[^19^F]ϕ	[^19^F]-ACI-87-Kϕ	*0.86	176	*1.26	140
D1-Q1Z-Kϕ	ACI-81-Kϕ	0.13	27	0.20	22
D1-Q1Z-H3F-Kϕ	ACI-84-Kϕ	0.31	63	0.58	64
D1-Q1X-Kϕ	ACI-82-Kϕ	0.82	167	1.28	142
D1-Q1X-H3F-Kϕ	ACI-85-Kϕ	0.63	129	1.00	111
D1-Q1X-V12K-Kϕ	ACI-88-Kϕ	*1.29	263	*1.78	198
D1-Q1X-V12K-K[^19^F]ϕ	[^19^F]-ACI-88-Kϕ	*1.05	214	*1.76	196
D1-Q1P-Kϕ	ACI-83-Kϕ	1.05	214	1.50	167
D1-Q1P-H3F-Kϕ	ACI-86-Kϕ	0.80	163	1.24	138
D1-Q1P-V12K-Kϕ	ACI-89-Kϕ	*1.06	216	*1.49	165
D1-Q1P-V12K-K[^19^F]ϕ	[^19^F]-ACI-89-Kϕ	*1.19	243	*1.86	207

All values were compared to that of ACI-80-Kϕ. Compound binding to compound film, containing predominantly monomers and to fibrils was measured. Average values of two or three experiments unless marked otherwise. *value of one single experiment only. Kϕ presents a lysine (K) linked to a fluorescein isothiocyanate (ϕ).

**Figure 2:**
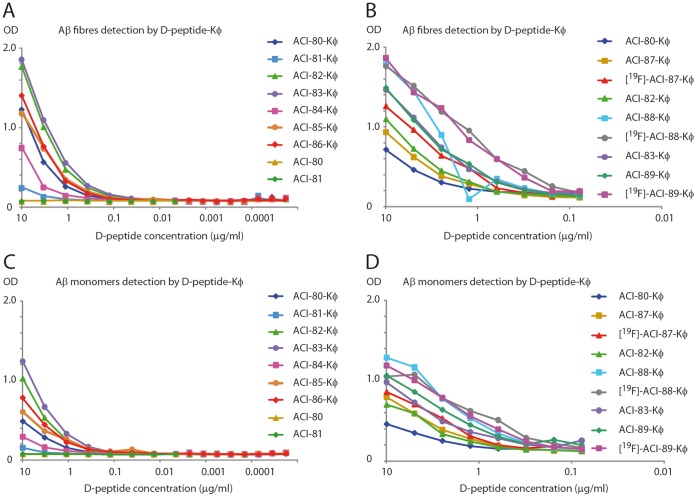
ELISA. Optical density (OD) at 450 nm measured at 2.5 h of pNPP incubation. OD for the different compounds at different concentrations is given. A and B illustrate the ability of the compounds to recognize Aβ fibrils. They indicate two series of experiments performed with following compounds A: ACI-80-Kϕ to ACI-86-Kϕ. ACI-80 without ϕ-label was run as a control. B: ACI-87-Kϕ to ACI-89-Kϕ, as well as fluorinated derivatives. ACI-80-Kϕ, ACI-82-Kϕ and ACI-83-Kϕ were included as controls. C and D illustrate the ability of the compounds to recognize peptide film which largely consists of Aβ monomers.

### Surface Plasmon Resonance: Single Cycle Experiments

Aβ1–42 fibrils were prepared as described above. Thereafter, the sample was centrifuged for 10 min at 16000×g, the supernatant discarded and this procedure repeated for 3 times. Formation of fibrils was confirmed by a standard Thioflavin-T fluorescence assay [26]. Aβ1–42 fibrils were covalently immobilized on a CM5 sensor chip via amine coupling. Prior to immobilization of Aβ1–42 fibrils the sample was centrifuged and fibrils resuspended in 10 mM sodium acetate buffer pH 4.0. Flow cell sensor surfaces were activated with a freshly prepared solution of 0.2 M 1-ethyl–3-(3-dimethylaminopropyl)-carbodiimide (EDC) and 0.05 M N-hydroxysuccinimide (NHS) at a constant flow rate of 10 µl/min for 420 s. Aβ1–42 fibrils (∼ 110 µM monomeric Aβ1–42) were injected for 600 s with a flow rate of 10 µl/min. Deactivation of the surface was performed by injection of 1 M ethanolamine-HCl pH 8.5 at the same flow rate and duration as in the activation step. In the reference cells the deactivation step was performed directly after the activation step.

**Figure 3:**
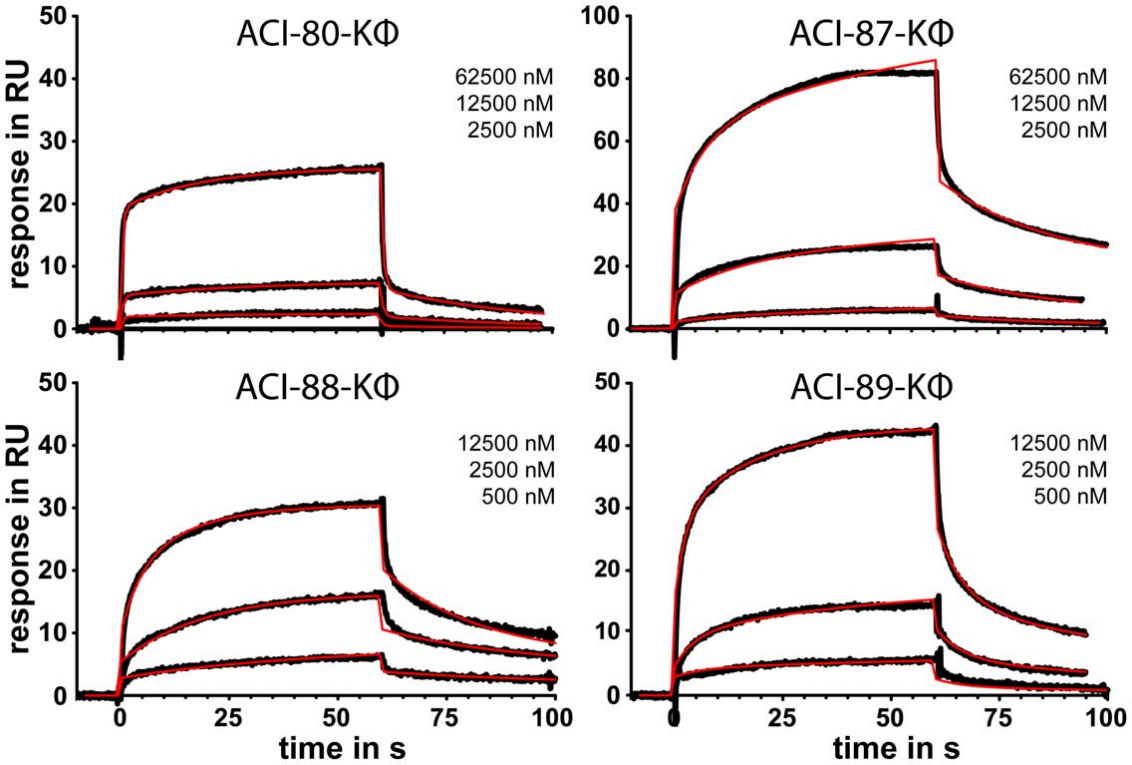
D-enantiomeric peptide variants binding to fibrillar Aß1–42, covalently immobilized on a CM5 sensor chip via amine coupling. For each peptide variant experimental sensorgrams (black traces) obtained with injections at 2500 nM, 12500 nM and 62500 nM (ACI-80-Kϕ, ACI-87-Kϕ) or 500 nM, 2500 nM and 12500 nM (ACI-88-Kϕ, ACI-89-Kϕ) are shown. Injections were performed for 60 seconds each and dissociation phases were recorded for at least 30 seconds. The sensorgrams were globally fit (red curves) to a heterogeneous ligand model accounting for different refractive indices.

**Table 5:** Results for compound – Aβ fibril interactions obtained with the heterogeneous ligand model.

Analyte	R_max_1	R_max_2	*k*_on_1	*k*_off_1	*k*_on_2	*k*_off_2	*K*_D_1	*K*_D_2
**ACI-80-Kϕ**	12.2	43.7	469	0.0252	1.37e4	1.58	5.38e-05	1.15e-04
**ACI-87-Kϕ**	43.3	36.2	206	1.84e-3	1.57e3	0.0407	8.93e-06	2.59e-05
**ACI-88-Kϕ**	9.96	63.3	1.71e4	7.22e-3	930	0.0553	4.22e-07	5.95e-05
**ACI-89-Kϕ**	21.8	19.4	2.03e4	0.162	3.34e3	0.0118	8.00e-06	3.53e-06

All SPR experiments were performed on a Biacore T100 system with series S CM5 sensor chips at 25°C. The system was run with the Biacore T100 Control Software Version 1.1.1. PBS (10 mM sodium phosphate buffer pH 7.4, 137 mM NaCl, 2.7 mM KCl) was chosen as running buffer (as previously during fibril formation) in order to minimize alterations of fibril organization. All buffers were sterile filtered (0.22 µm). After each docking of a sensor chip the detector was normalized with BIAnormalizing solution (70% glycerol, GE Healthcare) to compensate for slight differences in detector responses of individual sensor chips. For all interaction analyses the Type 1 reagent rack was used. Siliconized sample vials were used with their corresponding rubber caps (Type 2, GE Healthcare) to minimize evaporation effects. Throughout all runs the flow rate was set to 30 µl/min.

All interaction studies were performed in single-cycle mode [27]. Here, five different concentrations of analyte were passed through a reference cell and subsequently through flow cells with immobilized ligand within the same binding cycle for 60 seconds, starting with the lowest concentration. Successive injections were performed in the order of increasing concentrations. Each following concentration was a fivefold increase of the previous. The lowest analyte concentration was chosen to be 100 nM and therefore the following were 500, 2500, 12500 and 62500 nM.

**Figure 4:**
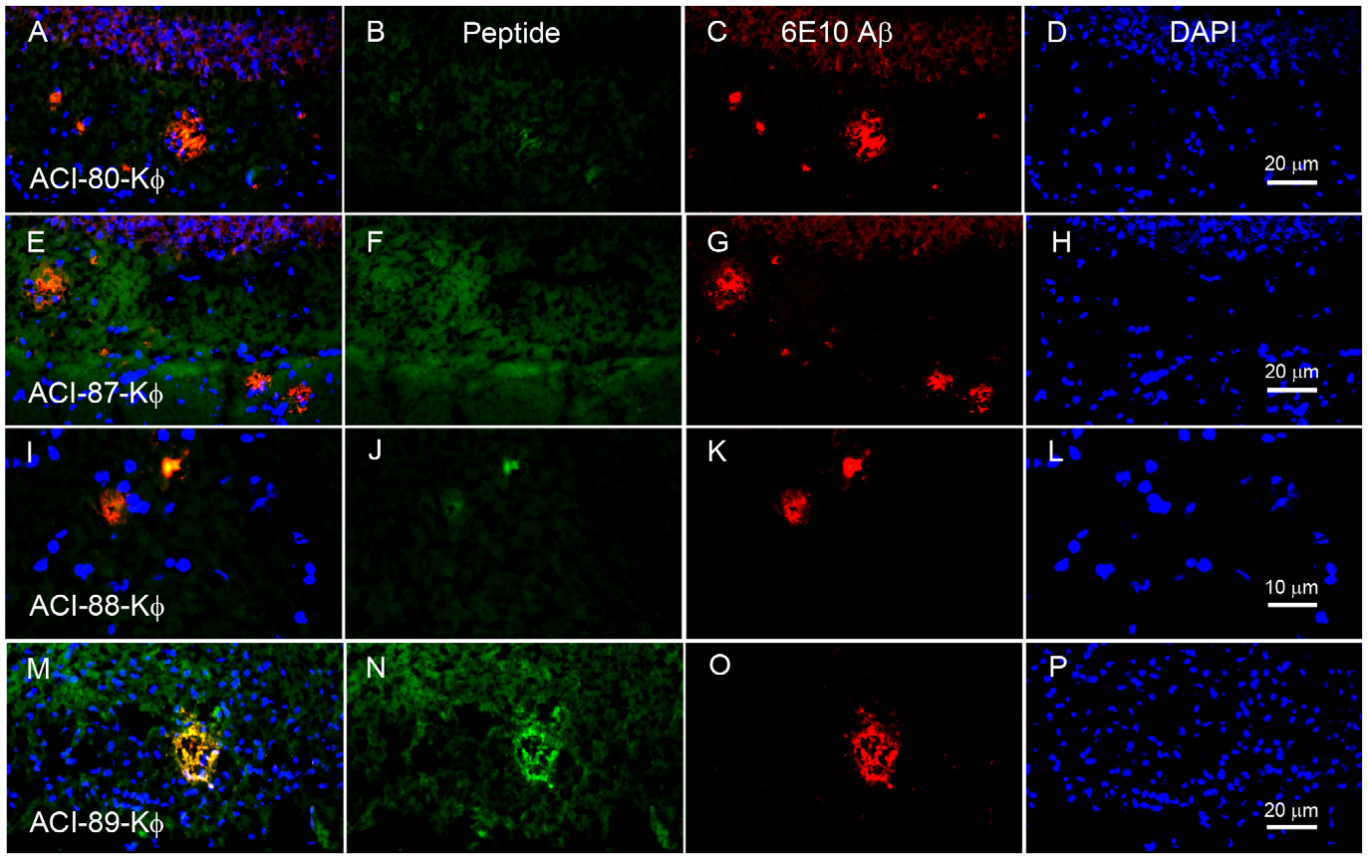
*Ex vivo* staining of brain tissue sections from 13 months old male double transgenic AD mice APP (V717I) x PS1 (A246E) using different ϕ-labeled ACI-80 derivatives, 6E10-Aβ-antibody and DAPI. Left column: triple image overlay of respective stains reveal that the ϕ-compounds identify plaques. White scale bars 20 µm. Results of non-transgenic litter mate controls are not shown as no staining of ϕ-labeled ACI-80 derivatives and 6E10-Aβ-antibody could be detected.

Biacore data were evaluated using BiaEvaluation 4.1.1 (GE Healthcare) and Biacore T100 Evaluation Software (GE Healthcare). Obtained binding data with compounds were double referenced. This was achieved by collecting the data in dual-channel mode with a reference flow cell connected upstream of the flow cell with immobilized Aβ1–42 fibrils and the subtraction of the obtained binding responses with a blank buffer injection (PBS). The double-referenced binding curves of the three lowest concentrations of each single cycle kinetics injection that showed a significant binding response were fit to a heterogeneous ligand binding model [28] including a factor correcting for different refractive indices (R_I_).

**Figure 5:**
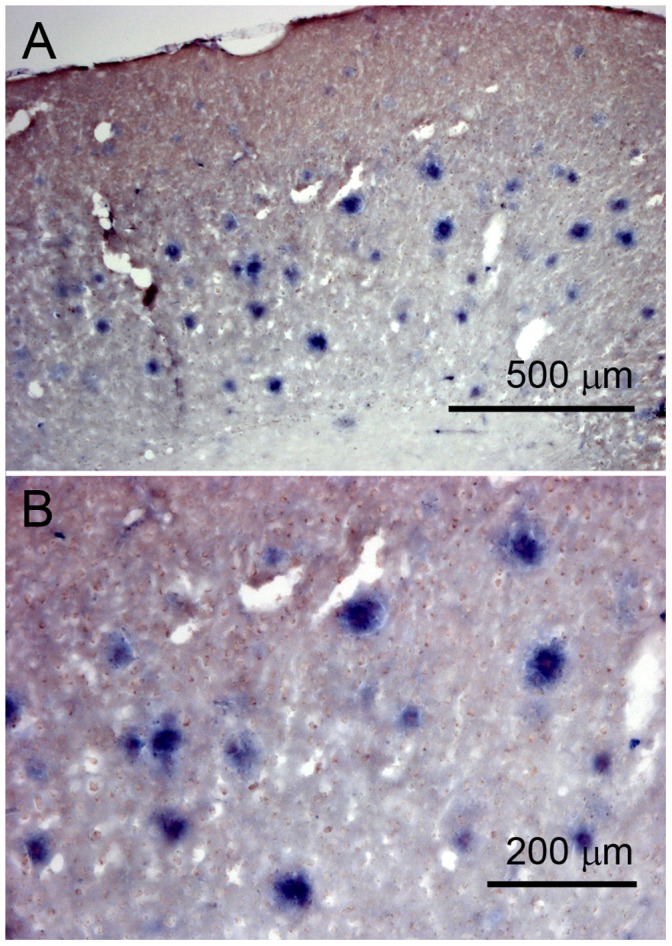
Photomicroscope images of mouse brain sections (female APP (V717I) × PS1 (A246E), age 24.3 months) in light microscope. Overview (left panel) and higher magnification (right panel). The brain slices were incubated with ACI-89-Kϕ-peptide binding to plaques was visualized using an anti-fluorecein isothiocyanate 1 antibody and alkaline phosphatase as chromogenic detection. This revealed the high sensitivity of this method and the presence of abundant plaques in the tg mouse brain.

### Enzyme-linked Immunosorbent Assays (ELISA)

#### Preparation of Aβ1–42 species

Aβ1–42 peptide film was prepared from lyophilized powder (Bachem). The powder was reconstituted in HFIP to a final concentration of 1 mM, sonicated for 15 min at room temperature (RT), agitated overnight (ON), and aliquoted into non-siliconized microcentrifuge tubes (12 µl corresponding to 55 µg). The HFIP was evaporated under a stream of argon. The resulting peptide film was vacuum dried for 10 min and stored at -80°C. For direct use as peptide film, an aliquot of Aβ1–42 peptide film was reconstituted with 0.54% dimethylsulphoxide (DMSO) in phosphate buffered saline (PBS) to obtain a final concentration of 10 µg/ml and then used for the ELISA as described below. To prepare fibrils, 55 µg aliquot of peptide film was dissolved in 55 µl of 50 mM Tris-HCl, pH 7.4 and incubated at 37°C for five days. Next, the sample was centrifuged (10’000 rpm for 5 min) and the pellet was diluted in 50 mM Tris-HCl, pH 7.4.

### D-peptide Compound Binding to Immobilized Aβ1–42

Aβ1–42 peptide preparations were diluted in 0.05 M bicarbonate-carbonate buffer pH 9.6, to the final concentration of 10 µg/ml and coated onto ELISA plates (MaxiSorp, Nunc). After blocking (PBS; 0.05% Tween; 1% BSA), plates were incubated with 2- or 3-fold dilutions of D-compounds (starting concentration: 10 µg/ml) and incubated for 2 h at 37°C. Plates were then washed and incubated for 2 h at 37°C with the detection antibody Rabbit-a-fluorescein isothiocyanate-AP (Sigma; 1∶10’000 dilution) followed by the incubation for 2.5 h at room temperature (RT) with 1 mg/ml of phosphatase substrate (pNPP, Sigma). The absorbance signal was read at 405 nm wavelength using a Tecan plate reader (Tecan Group Lt, Männedorf, Switzerland).

### D-peptide Compound Binding to Aβ1–42 in Solution

ELISA plates (MaxiSorp, Nunc) were coated with anti-Aβ antibody 6E10 (Covance) at a concentration of 5 µg/ml. Either monomeric Aβ1–42 peptide film (mainly monomeric) or Aβ1–42 fibrils, which were prepared as described above, were diluted in PBS to the final concentration 10 µg/ml and mixed with 10-fold dilutions of D-peptide compounds in Eppendorf tubes (starting concentration of D-peptide compounds: 10 µg/ml). The tubes were incubated for 2 h at 37°C. Next, samples were distributed onto the ELISA plate and kept for 2 h at 37°C. Plates were washed and incubated for 2 h at 37°C with the detection antibody Rabbit-a-fluorescein isothiocyanate-AP (Sigma; 1∶10′000 dilution) following the incubation overnight (ON) at RT with 1 mg/ml of phosphatase substrate (pNPP, Sigma). The absorbance signal was read at 405 nm wavelength using the Tecan plate reader (Tecan Group Ltd, Männedorf, Switzerland).

### Ex vivo Staining of Mouse Brain Slices by FITC Labeled Compounds

*In vitro* tissue section staining was performed according to previously described protocols [20] with slight modifications. Mouse brains were obtained from male transgenic (tg) APP (London mutation V717l) x PS1 (A246E) mice aged 13–21 months and from female wild type (wt) mice aged 9–10 months [29]. The mice were anesthetized and transcardially perfused with saline. The brains were removed and snap frozen. 10 µm thick sagittal cryostat sections through the whole mouse brain were produced and mounted onto glass slides. ϕ-labeled compounds were applied to investigate binding. Thus, the slides were thawed, washed in PBS and fixed in 4% paraformaldehyde (20 min at RT) just before incubation with ϕ-labeled compounds. One series of sections was treated only with fluorescent compounds (0.01 mg/ml, incubation time 2 h) while another series of sections were incubated with anti-Aβ antibody 6E10 at 1∶500 dilution (SIG-39320, Covance; final antibody concentration was 2 µg/ml) in addition to ϕ-labeled compounds (0.01 mg/ml). The slices were washed in PBS. The sections incubated with ϕ-labeled compound and 6E10 antibody were further incubated for 2 h at RT with Goat-anti-Mouse IgG1– AlexaFluor 555 (A21127, Invitrogen, 1∶1000 dilution) and washed with PBS. Finally, all sections were counterstained with 4’–6-Diamidino-2-phenylindole (DAPI, 32670, Sigma, incubation 5 min at RT) for the visualization of cell nuclei. The slides were mounted using Prolong Gold Antifade mounting medium (P36930, Invitrogen) and coverslipped.

The tissues were analyzed using a fluorescent Zeiss Axioscope 2 Plus microscope using the AxioVision 4 image analysis software.

A further series of slices was stained using a primary anti-fluorescein isothiocyanate antibody combined with alkaline phosphatase reaction for visualization of ϕ-labeled peptides. Briefly, the sections were treated with blocking solution (10% normal goat serum (NGS), 0.25% Triton X-100 in PBS for 1 h at RT) and incubated with primary antibody (rabbit-anti-FITC; Invitrogen) at a dilution of 1∶500. The sections were then washed and incubated for 2 h at RT with the secondary antibody goat-anti-rabbit-alkaline phosphatase (Sigma) at a dilution of 1∶100. After washing, the slides were incubated with BCIP/NBT substrate (Sigma) for 3 min, washed, dehydrated and mounted using Eukitt mounting medium.

## Results

### Analysis of ACI-80 Amino-terminal Residue Identity

An important observation during initial compound stability characterization experiments was the partial conversion of the N-terminal glutamine of [^127^I]-ACI-80 into pyro-glutamate. It is known that peptides with an N-terminal glutamine are prone to conversion of this residue into a pyroglutamate [30]. Therefore, the composition of freshly synthesized and untreated ACI-80 and ACI-80-Kϕ as well as [^127^I]-ACI-80 after iodination were investigated. The results are displayed in Table 1. It was observed that freshly synthesized ACI-80 and ACI-80-Kϕ contained only minor fractions of pyroglutamic acid while about one third of [^127^I]-ACI-80 in solution already converted into N-terminal pyroglutamate species.

Thus, a number of novel ACI-80-derivatives were designed and synthesized with one or several amino acid deletions and/or substitutions with the aim to increase compound stability, but also Aβ binding capability. Specifically, the N-terminal glutamine was either substituted by pyroglutamate (Q1Z) or proline (Q1P) or deleted (Q1X). Furthermore, a replacement of His-3 by phenylalanine (H3F) was investigated. The Q1X deletion as well as Q1P and H3F substitutions have been proposed based on semiquantitative saturation mutagenesis peptide spot data, which predicted that the mutations would enhance Aβ binding. In the peptide spot approach, all amino-acids of ACI-80 were substituted against all other natural amino acids and the variants were tested for their ability to bind Aβ fibrils (data not shown). In addition valine was replaced by lysine at position 12 (V12K) to enable fluorination of the compound for imaging purposes. Finally, the compound was also fluorescein isothiocyanate (ϕ)-labeled via an additional C-terminal lysine residue to enable detection through fluorescence or via anti-fluorescein isothiocyanate antibodies. For a summary of all ACI-80 derivatives see Table 2.

### Surface Plasmon Resonance and ELISA

To characterize the ACI-80 derivatives in respect to their Aβ binding capabilities in comparison to ACI-80-Kϕ, a number of *in vitro* assays were performed.

Surface plasmon resonance (SPR) assays were performed to analyze their interaction with immobilized Aβ1–42 fibrils (Table 3). Aβ1–42 fibrils were immobilized on a Biacore sensor-chip as described in the methods section, and the interactions of the ϕ-labeled ACI-80 derivatives were measured and compared with original ACI-80-Kϕ. Due to the fact that Aβ1–42 fibrils represent an inhomogeneous mixture of different fibril aggregates, it is hardly possible to form a homogenous Aβ1–42 loaded SPR chip surface. Therefore we decided for semi-quantitative comparison between all compounds only. The maximal responses in resonance units (RU) of the ϕ-labeled ACI-80 derivatives during the analyte injection and the remaining response 60 s after injection end (as a semi-quantitative measure for the dissociation rate), have been related to the respective values obtained from identical concentrations of ACI-80-Kϕ in percent. The results are summarized in Table 3 and Figure 1.

ACI-80-Kϕ derivatives ACI-82-Kϕ and ACI 83-Kϕ (aa modifications Q1X and Q1P) yielded an increased response of up to 600% as compared to ACI-80-Kϕ (Table 3). The response for ACI-81-Kϕ (aa substitution Q1Z) was not significantly increased. The substitution at the third amino acid position (H3F) did not lead to improved binding to Aβ. While ACI-84-Kϕ behaved slightly worse than ACI-81-Kϕ, the binding efficiencies of ACI-85-Kϕ and ACI-86-Kϕ were drastically reduced as compared with those of ACI-82-Kϕ and ACI-83-Kϕ, respectively. The single substitution at aa position 12, valine to lysine (ACI-87-Kϕ), resulted in a response increase to more than 300%. Compounds ACI-88-Kϕ and ACI-89-Kϕ with two substitutions, the first one at amino acid position 1 (Q1X, Q1P) and the second one at amino acid position 12 (V12K), yielded a response increase to 350% (Q1X-V12K) and more than 500% (Q1P-V12K). The substitution V12K had an important impact with respect to dissociation. All three compounds with a V12K substitution showed decreased dissociation rates (increased remaining response 60 s after injection end) as compared to the compounds without the V12K substitution. Therefore, ACI-87-Kϕ, ACI-88-Kϕ, and ACI-89-Kϕ were the most promising compounds for further studies. In order to investigate, whether [^18^F]-labeling will change their binding affinities, the respective [^19^F]-labeled compounds have been investigated as well. Except for [^19^F]-ACI-87-Kϕ (Q1X-V12K), which showed a decreased response in comparison to the compound without label, the [^19^F]-labeled compounds showed virtually the same behavior with respect to maximum binding. The remaining response 60 s after injection end, however, was significantly changed in all three cases. Based on the remaining response 60 s after injection end, [^19^F]-ACI-88-Kϕ exerted the slowest off-rate of all tested compounds indicating the most suitable binding behavior for being used as a molecular probe.

To verify the SPR results, ELISA was employed to assay the interaction of the ACI-80 derivatives ACI-80-Kϕ, ACI-81-Kϕ, ACI-82-Kϕ, ACI-83-Kϕ, ACI-84-Kϕ, ACI-85-Kϕ, ACI-86-Kϕ, ACI-87-Kϕ, [^19^F]-ACI-87-Kϕ, ACI-88-Kϕ, [^19^F]-ACI-88-Kϕ, ACI-89-Kϕ, [^19^F]-ACI-89-Kϕ) with Aβ1–42 fibrils and Aβ peptide film in solution containing mostly monomers and smaller oligomers (Table 4). To avoid any bias by possible conformational influences the ELISA has been carried out in two versions, once with immobilized antibody and once with immobilized Aβ1–42 as described in the methods section. Both assays yielded similar results and confirmed each other. All experiments were performed twice and showed reproducible results. Panels A and B in Figure 2 show the results for binding to Aβ1–42 fibrils and panels C and D in Figure 2 show the results for binding to freshly prepared Aβ1–42 peptide film, which contains mostly monomeric Aβ. In Table 4, the performance of the peptides is expressed relative to that of ACI-80-Kϕ.

Briefly summarized, the binding of the ACI-80 derivatives to Aβ1–42 fibrils was generally stronger than that to monomer-enriched freshly prepared Aβ1–42. In addition, for both Aβ species a similar order of binding strengths could be established. All variants, except ACI-81-Kϕ and ACI-84-Kϕ, showed stronger Aβ binding than ACI-80-Kϕ. Substitution of glutamine to proline at position 1 or glutamine deletion had a positive effect on binding to Aβ1–42. Inversely, binding to Aβ1–42 was reduced for ACI-84-Kϕ as compared to ACI-80-Kϕ. ACI-81-Kϕ and ACI-84-Kϕ are peptides with glutamine to pyroglutamate substitution. Thus, the substitution of glutamine to pyroglutamate decreased binding to Aβ1–42. An order of binding comparing fluorinated with respect to non-fluorinated D-peptides versions was difficult to establish. The results clearly show that the fluorinated ϕ-labeled peptides [^19^F]-ACI-87-Kϕ, [^19^F]-ACI-88-Kϕ, [^19^F]-ACI-89-Kϕ bound well to Aβ1–42 fibrils, [^19^F]-ACI-88-Kϕ, [^19^F]-ACI-89-Kϕ being even among the very best of all variants. The order of binding strengths for the fluorinated peptides was [^19^F]-ACI-89-Kϕ binds stronger than [^19^F]-ACI-88-Kϕ much stronger than [^19^F]-ACI-87-Kϕ. These peptides with [^19^F] replaced by [^18^F] were used in autoradiography assays for testing the binding to human Alzheimer’s brain tissue sections [24]. Briefly, the experiments, using *post mortem* human brain autoradiography in whole hemisphere human brains obtained from deceased AD patients and age matched control subjects, support the visualization capacity of the radiolabeled ACI-80 analogues of amyloid deposits in the human brain [24].

In general, the ELISA results corresponded well with the SPR results identifying the substitution of glutamine to proline or glutamine deletion at the N-terminus as useful mutation with strong benefits for the binding to Aβ fibrils and monomers.

As the compounds ACI-87-Kϕ, ACI-88-Kϕ, ACI-89-Kϕ were the most promising candidates for further development, we tried to obtain more quantitative binding data and compared them to ACI-80-Kϕ. Different concentrations of the analytes were applied to an SPR chip loaded with Aβ fibrils (Fig. 3). A brief look at the data already revealed that about a five-fold concentrations of ACI-87-Kϕ and ACI-80-Kϕ were necessary to obtain comparable RU responses as compared to ACI-88-Kϕ and ACI-89-Kϕ. All three ACI-80 derivatives showed tighter binding to fibrils as compared to ACI-80-Kϕ. As already mentioned above, any effort to obtain an exact quantitative analysis of experimental data from SPR experiments with Aβ fibrils is prone to mis- and over-interpretation. We found, however, that the heterogeneous ligand binding model (see methods section for details) was able to yield potentially meaningful results for all four compounds without introducing too many fit parameters. With all necessary caution and taking into account only the lowest *K*_D_ value of the two obtained from the heterogeneous binding model, the fitted *K*_D_ values given in Table 5 confirm the order of binding: with an obtained dissociation constant in the submicromolar range ACI-88-Kϕ binds stronger than ACI-89-Kϕ and ACI-87-Kϕ, and all three of them bind stronger than ACI-80-Kϕ.

### Ex vivo Staining of Mouse Brain Slices by FITC Labeled Compounds

Brain sections from transgenic (tg) APP (London mutation V717l) x PS1 (A246E) mice were stained using ACI-80-Kϕ, ACI-87-Kϕ, ACI-88-Kϕ, ACI-89-Kϕ. Also, anti-Aβ plaque staining (using the 6E10 antibody) and DAPI nuclei counterstaining was performed on the same slides. Photomicrographs of the stained slices and triple overlay images are shown in Figure 4.

Aβ plaques were identified in all tg animals using 6E10 anti-Aβ antibody. Moreover, plaques were stained by all tested compounds, although to different extent and with different intensity and background. Specifically, ACI-89-Kϕ and ACI-88-Kϕ stained plaques most intensively, whereas ACI-88-Kϕ additionally yielded the lowest background signal of all peptides. As shown in Figure 4, ACI-89-Kϕ and ACI-88-Kϕ match 6E10 anti-Aβ staining quite well giving rise to a large extent of overlay in the triple exposure.

For the peptide ACI-87-Kϕ, nearly no overlay with 6E10 anti-Aβ was detected by means that only a very small fraction of the plaques identified with anti-Aβ antibody 6E10 have been stained with the D-peptide. The detection of overlay by the eye is additionally hindered by high background fluorescence. Moreover, ACI-89-Kϕ and ACI-88-Kϕ showed a slightly different staining pattern by means that ACI-88-Kϕ stained the core of plaques while ACI-89-Kϕ has a staining pattern which is more similar to the one of 6E10 which also stains diffuse Aβ plaques.

Therefore, the qualitative assessment demonstrates that the peptides are able to recognize plaques in tg mouse brains with different intensities and background signals. Overall, [^19^F]-ACI-89-Kϕ and ACI-88-Kϕ showed a good overlap with 6E10 Aβ staining and have thus confirmed their leading roles as candidates for further evaluation.

To further proof with a fluorescent independent read-out that ACI-89-Kϕ binds to plaques after peripheral injection, an antibody recognizing the FITC group in ACI-89-Kϕ was used (Figure 5). This antibody binds to ϕ-labeled peptides that are bound to Aβ plaques in ϕ-peptide immersed tg mouse brain slices. The alkaline phosphatase reaction showed abundant chromogenic deposits resembling the expected distribution and number of Aβ plaques in these brains identified by ACI-89-Kϕ. Thus confidence was provided that ACI-89-Kϕ binds to brain Aβ plaques following peripheral application.

## Discussion

One of the hallmarks of AD is Aβ accumulation in plaques, probably long time before manifestation of clinical symptoms. Here, we have characterized novel ligands that we believe can have the potential to be used for diagnostic imaging in patients with AD and also in individuals that score as MCI. There is strong demand for imaging probes that allow early diagnosis of the disease, thus enabling novel therapies that allow early intervention. Also such probes will be important to monitor disease progression and therapy success in longitudinal studies. The ligands also have the potential to be used for PET imaging, for example in transgenic mouse models, that overexpress the amyloid precursor proteins and develop amyloid plaques, or in aged monkeys. Imaging of parenchymal Aβ plaques, which mainly consist of the isoform Aβ1–42 in both, transgenic animals and humans, heavily relies on molecular probes that are specifically binding to Aβ1–42 fibrils. In order to discriminate between the two most relevant Aβ depositions in AD, namely vascular Aβ, which mainly consists of Aβ1–40, and parenchymal Aβ, which mainly contains Aβ1–42, there is an urgent need for such a specific PET ligand, as it is not clear if the currently most advanced [^11^C]-PIB-PET compound discriminates between Aβ1–40 and 1–42 *in vivo* [31].

The lead compounds ACI-87-Kϕ, ACI-88-Kϕ and ACI-89-Kϕ of this program were derived from ACI-80, which is a D-enantiomeric, 12 amino acid peptide that originally was selected by mirror-image phage display (24). D-peptides have several advantages over L-enantiomeric peptides. Most importantly, they are resistant to most proteases [32], which can dramatically increase serum [33] and saliva [34] half-life.

The need for exploring derivatives of ACI-80 was dictated by the observation that the N-terminal amino acid residue of ACI-80 converted from glutamine to pyroglutamate in aqueous solution. In addition, ACI-80 derivatives with increased binding affinity to aggregated Aβ species were desirable. The lead compounds ACI-87-Kϕ, ACI-88-Kϕ and ACI-89-Kϕ were stable in aqueous solution and showed even superior Aβ binding characteristics as compared to ACI-80-Kϕ. This was confirmed by ELISA and SPR *in vitro* binding assays. The ELISA results were fully compatible with the results from SPR. In general, a stronger binding of ACI-80-Kϕ and its derivatives to aggregated Aβ forms, in comparison to monomeric forms, could be verified by ELISA. This is in accordance to the observation previously reported for ACI-80 [25]. Whether the ACI-80 derivatives also inherited the ACI-80 property to preferentially bind Aβ1–42 over Aβ1–40, was not investigated in the present study.

All *in vitro* binding data agree that Q1X and Q1P mutations lead to an increase of binding and a decrease of dissociation rate, whereas the H3F mutation led to a decrease in binding. In line with the SPR results, the ELISA data confirmed that the substitution of glutamine to proline and the glutamine deletion increased binding to Aβ1–42 whereas the substitution of glutamine to pyroglutamate decreased binding to Aβ. Also, fluorinated, ϕ-labeled D-compounds bound well to Aβ fibrils.

The SPR measurements that were carried out to compare the binding capabilities of ACI-80-Kϕ, ACI-87-Kϕ, ACI-88-Kϕ and ACI-89-Kϕ to Aβ-fibrils once more confirmed the binding order: ACI-88-Kϕ binds stronger than ACI-89-Kϕ stronger than ACI-87-Kϕ much stronger than ACI-80-Kϕ. Although not all of the fitted binding curves do perfectly fit to the experimental data, the applied evaluation procedure yielded some values for binding affinities that allowed comparison between the four compounds.

*Ex vivo* staining of transgenic mouse brains showed that the FITC labeled compounds ACI-87-Kϕ, ACI-88-Kϕ and ACI-89-Kϕ and their fluorinated derivatives [^19^F]-ACI-87-Kϕ, [^19^F]-ACI-88-Kϕ, and [^19^F]-ACI-89-Kϕ readily recognized amyloid plaques in the mouse brain sections. This is important evidence that these compounds can be used to monitor therapy progress in AD mouse models. Interestingly, [^19^F]-ACI-89-Kϕ showed a different staining pattern in comparison to the other compounds, being more diffuse and comparable to the staining of 6E10 Aβ antibody.

As already described in two other reports [23], [24] positive autoradiography (ARG) signals, compatible with Aβ staining, have been found in cortical gray matter using [^18^F]-ACI-87-Kϕ, [^18^F]-ACI-88-Kϕ, [^18^F]-ACI-89-Kϕ, [^125^I]-ACI-80 and [^125^I]-ACI-80-Kϕ ARG on human whole hemisphere brain sections of patients with AD. Brain sections from non-Alzheimer’s control subjects were significantly less stained in the cortical gray matter, underpinning the specificity of the ARG signal.

In conclusion, especially the ACI-80 derivatives ACI-87-Kϕ, ACI-88-Kϕ and ACI-89-Kϕ show superior binding affinities and specificities suggesting them as potential probes for specific Aβ aggregate and plaque detection in the living brain.
